# Pancreatic Heterotopia Presenting as Ileal Intussusception in an Adult: A Case Report and Literature Review

**DOI:** 10.7759/cureus.86745

**Published:** 2025-06-25

**Authors:** Noorah Alhosani, Ahmad Al Rifai

**Affiliations:** 1 Gastroenterology and Hepatology, Sheikh Shakhbout Medical City, Abu Dhabi, ARE

**Keywords:** acute abdomen, ectopic pancreas, ileo-ileal intussusception, pancreatic heterotopia, small-bowel intussusception, small intestinal obstruction, unexplained abdominal pain

## Abstract

Pancreatic heterotopia is a rare congenital disorder characterized by ectopic pancreatic tissue without anatomical or vascular connection to the native pancreas. It is mostly asymptomatic and can present with non-specific gastrointestinal symptoms. Rare complications such as intussusception have been reported with this condition.

We report the case of a 38-year-old female with a history of intermittent abdominal pain that was initially labeled and managed as irritable bowel syndrome. Advanced imaging revealed small intestinal intussusception, which turned out to be secondary to pancreatic heterotopia. The diagnosis was confirmed postoperatively through histopathological examination. Recent studies indicate that while the incidence of pancreatic heterotopia is relatively low, it is frequently identified as an incidental finding during abdominal surgeries, with intussusception reported in rare cases as a lead point for obstruction. Imaging modalities such as contrast-enhanced CT and MRI are pivotal for diagnosis, although laparotomy often remains essential for definitive management. This case highlights the diagnostic challenges and underscores the importance of advanced imaging techniques and multidisciplinary management in resolving atypical abdominal presentations.

## Introduction

Pancreatic heterotopia is a rare congenital anomaly, defined as the presence of ectopic pancreatic tissue that is anatomically and functionally independent of the pancreas. This condition results from embryological malformations during foregut rotation, leading to pancreatic tissue being deposited in aberrant locations within the gastrointestinal tract, most commonly the stomach, duodenum, and jejunum. The reported prevalence ranges from 0.5% to 13.7% based on surgical and autopsy series [1-3]. While many cases remain asymptomatic and are diagnosed incidentally during abdominal surgeries, heterotopic pancreatic tissue can manifest clinically with non-specific symptoms such as abdominal pain, nausea, vomiting, and gastrointestinal bleeding, depending on the lesion's size, location, and associated complications [3,4].

A particularly rare but significant complication of pancreatic heterotopia is small bowel intussusception, where the ectopic tissue acts as a lead point for obstruction. Such cases pose diagnostic challenges due to overlapping symptoms with other gastrointestinal conditions and the limitations of imaging modalities in detecting small, submucosal lesions. Advanced imaging techniques, including contrast-enhanced CT and MRI, are increasingly recognized as important tools for diagnosis, although definitive diagnosis often relies on histopathological evaluation following surgical resection [5,6].

This report presents the case of a 38-year-old female with chronic intermittent abdominal pain, ultimately diagnosed with small intestinal intussusception caused by pancreatic heterotopia. This case underscores the importance of maintaining a high index of suspicion for this rare condition, particularly in patients with recurrent, unexplained abdominal symptoms, to facilitate timely diagnosis and management.

## Case presentation

A 38-year-old female with a medical history significant for migraines presented to the ED with persistent abdominal pain. The patient had a history of intermittent abdominal pain for more than one month, with multiple visits to different EDs. The pain was described as gradual in onset, colicky in nature, and diffusely located in the periumbilical region. It was not associated with nausea, vomiting, diarrhea, fever, or overt gastrointestinal bleeding. The patient's symptoms were documented to be exacerbated by anxiety, and she reported no identifiable relieving factors. Initial physical examination revealed a soft, non-tender abdomen with no palpable masses, and her vital signs were stable. Laboratory investigations, including inflammatory markers, liver enzymes, and serum lipase levels, were unremarkable (Table 1). She received symptomatic treatment and was discharged from the ED with a presumptive diagnosis of irritable bowel syndrome (IBS) and referred to the gastroenterology clinic.

**Table 1:** Relevant laboratory investigations ALP, alkaline phosphatase; ALT, alanine aminotransferase; AST, aspartate transaminase; CRP, C-reactive protein; FIT, fecal immunochemical test; WBC, white blood cell

Test	Result	Reference range
WBC	5.24x10^9^/L	4-11x10^9^/L
CRP	8.76 mg/dL	≤5 mg/dL
AST	12 IU/L	≤32 IU/L
ALT	8 IU/L	≤33 IU/L
ALP	52 IU/L	35-104 IU/L
Albumin	33 g/L	35-52 g/L
Lipase	38 U/L	≤60 U/L
Calprotectin (stool) quantitative	529.57 μg/g	0-49.9 μg/g
FIT	11 μg/g	0-9.9 μg/g

Over the subsequent three weeks, the patient presented to the ED multiple times with worsening abdominal pain. During one of these visits, a CT scan of the abdomen was performed, which did not show any cause for the pain. Her symptoms persisted, prompting further evaluation, including fecal calprotectin, which was elevated at 567 μg/g, and a positive fecal immunochemical test (FIT) (Table 1). In view of those positive results, the patient underwent an upper GI endoscopy and a colonoscopy. The upper GI endoscopy revealed antral erosions with *Helicobacter pylori* positivity, while colonoscopy showed no significant findings. She was successfully treated for *H. pylori* gastritis; however, her symptoms persisted and remained unexplained.

**Figure 1:**
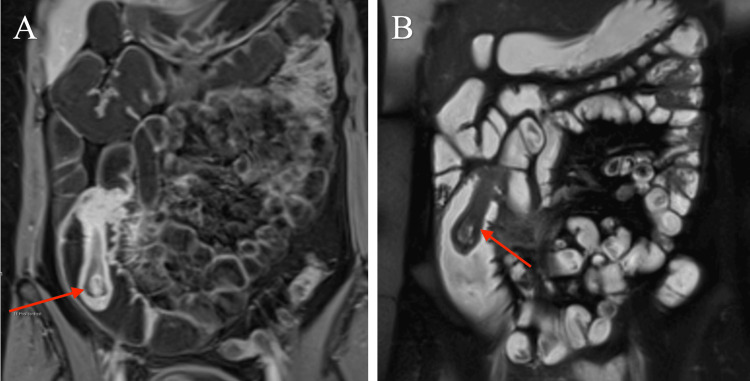
(A) MRI abdomen after contrast showing enhancing polypoidal mass at the terminal ileum (red arrow). (B) Coronal T2 MRI showing dilated terminal ileum and the polyp (red arrow)

**Figure 2:**
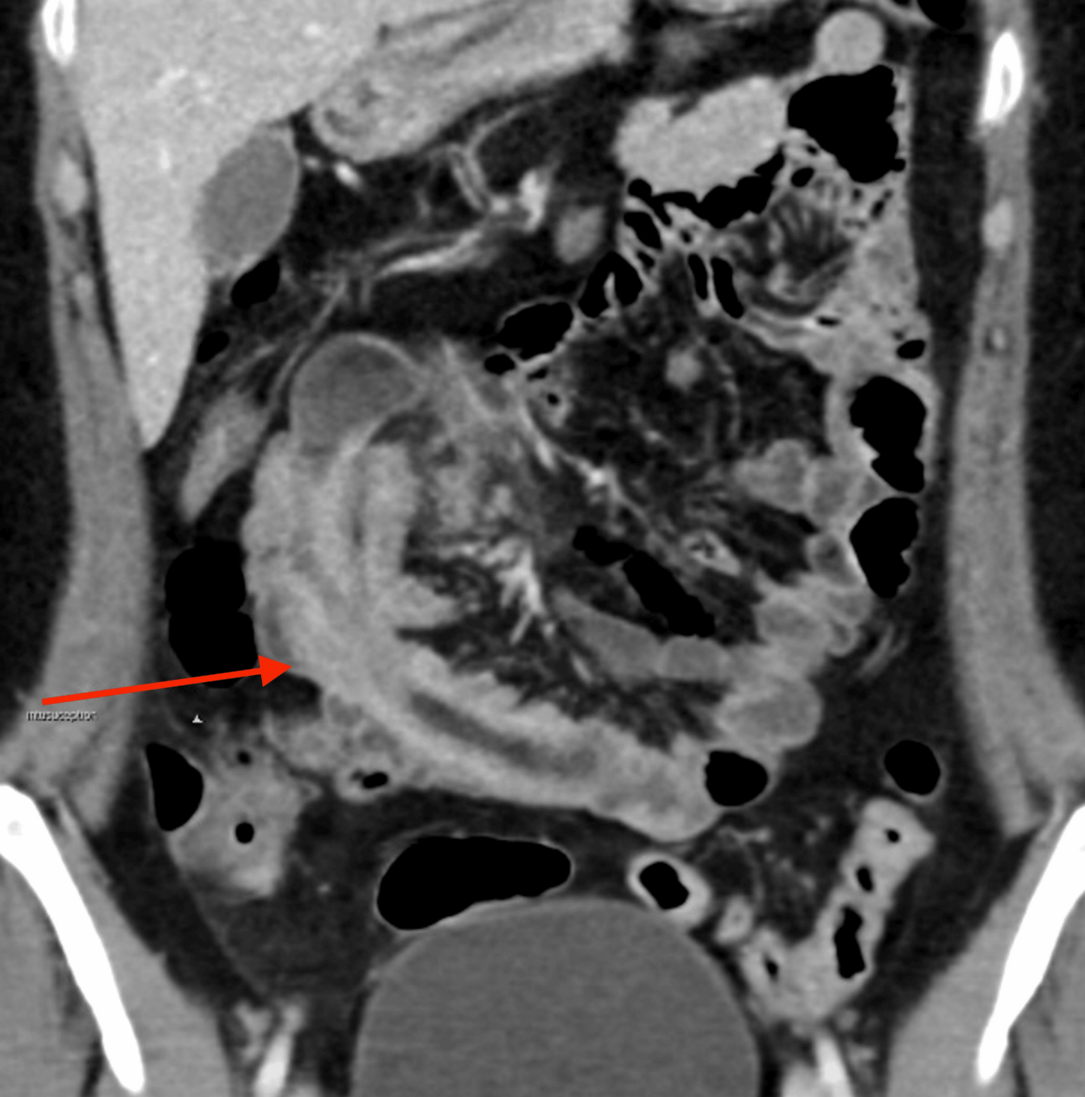
Coronal CT abdomen after contrast showing intussusception with a lead point (red arrow)

On follow-up in the gastroenterology clinic, the patient reported ongoing colicky abdominal pain, now associated with weight loss of 2 kg, a history of black stools, and intermittent episodes of hematochezia. An urgent MRI of the abdomen with contrast was arranged. This revealed a 2×5 cm polypoidal mass projecting into the cecum and associated with ileocecal intussusception (Figure 1). No evidence of acute colitis or enteritis was observed. The patient was referred to the general surgery department for further management; however, due to the ongoing pain and the findings on MRI, she was admitted, and a CT scan was performed urgently. The CT scan demonstrated ileo-ileal intussusception involving a 10 cm segment of the small bowel with unclear underlying pathology (Figure 2).

She underwent an emergency laparotomy, during which a 10-cm segment of small bowel intussusception located 60 cm proximal to the ileocecal junction was identified and successfully resected. The resected specimen was sent for histopathological examination, which revealed pancreatic heterotopia as the underlying etiology.

The patient’s postoperative recovery was uneventful, and she was discharged within a few days in stable condition. At her two-month follow-up visit, she remained asymptomatic with no recurrence of her symptoms.

## Discussion

Pancreatic heterotopia is a rare congenital anomaly characterized by the presence of pancreatic tissue in an ectopic location, lacking anatomical and vascular continuity with the native pancreas. The underlying embryological mechanism is not fully understood but is thought to involve aberrant migration during foregut development [1-3]. While the stomach, duodenum, and proximal jejunum account for over 90% of reported cases, occurrence in the ileum, as observed in this case, is uncommon and rarely described in the literature [6,7].

The majority of heterotopic pancreas cases are asymptomatic and diagnosed incidentally during surgical procedures or imaging performed for unrelated reasons. When symptoms are present, they are typically nonspecific and may include abdominal pain, nausea, vomiting, or GI bleeding [3,4]. One rare but clinically significant complication is small bowel intussusception, where ectopic pancreatic tissue can serve as a pathological lead point [4]. Due to its infrequent nature and variable presentation, diagnosis can be delayed or missed, particularly in the absence of overt obstructive symptoms.

Histologically, pancreatic heterotopia is defined by the presence of pancreatic acini, ducts, and islet cells at an ectopic site [8]. Although generally benign, this tissue is susceptible to the same pathological changes as the orthotopic pancreas, including inflammation, cyst formation, and rare malignant transformation [3,9]. 

In the present case, the patient experienced intermittent abdominal pain over several weeks and underwent multiple ED evaluations. Initial laboratory investigations and cross-sectional imaging, including CT, were unremarkable, and the clinical picture was initially attributed to IBS. Subsequent evaluation with fecal biomarkers yielded elevated calprotectin and a positive FIT (Table 1), prompting endoscopic assessment. Endoscopy did not reveal a cause for the patient’s symptoms.

Further evaluation with MRI identified a polypoidal lesion at the terminal ileum associated with ileocecal intussusception. The ileo-ileal intussusception was diagnosed later on an urgent CT that was performed due to worsening of symptoms. Surgical exploration and histopathological examination ultimately revealed pancreatic heterotopia as the underlying etiology.

This case underscores the diagnostic complexity associated with heterotopic pancreas, particularly when the ectopic tissue is located in an atypical site such as the distal ileum. Standard imaging modalities may fail to detect small or submucosal lesions, and the nonspecific nature of presenting symptoms can lead to misdiagnosis [9]. Management of pancreatic heterotopia is guided by the clinical presentation. Asymptomatic lesions may be managed conservatively, while symptomatic or complicated cases, including those involving intussusception, typically require surgical resection [4,9]. In this case, segmental small bowel resection led to complete resolution of symptoms. Long-term follow-up may be considered in conservatively managed patients to monitor for potential complications.

## Conclusions

This case highlights the importance of thinking about pancreatic heterotopia and small bowel diseases in the differential diagnosis of recurrent, unexplained abdominal pain, particularly when standard investigations are inconclusive. Awareness of its atypical presentations and potential complications is essential to ensure timely diagnosis and appropriate management.
